# Development of Immunoassays for Detection of *Francisella tularensis* Lipopolysaccharide in Tularemia Patient Samples

**DOI:** 10.3390/pathogens10080924

**Published:** 2021-07-22

**Authors:** Emily E. Hannah, Sujata G. Pandit, Derrick Hau, Haley L. DeMers, Kayleigh Robichaux, Teerapat Nualnoi, Anjana Dissanayaka, Jose Arias-Umana, Heather R. Green, Peter Thorkildson, Kathryn J. Pflughoeft, Marcellene A. Gates-Hollingsworth, Yasemin Ozsurekci, David P. AuCoin

**Affiliations:** 1 Department of Microbiology and Immunology, Reno School of Medicine, University of Nevada, Reno, NV 89509, USA; em.hannah93@gmail.com (E.E.H.); spandit@med.unr.edu (S.G.P.); hau.derrick@gmail.com (D.H.); kinney.hl@gmail.com (H.L.D.); leighjordan17@gmail.com (K.R.); teerapat.n@psu.ac.th (T.N.); anjanad90@gmail.com (A.D.); jariasumana@nevada.unr.edu (J.A.-U.); hrg@nevada.unr.edu (H.R.G.); pthorkildson@med.unr.edu (P.T.); kpflughoeft@med.unr.edu (K.J.P.); mhollingsworth@med.unr.edu (M.A.G.-H.); 2 Faculty of Medicine, Hacettepe University, Ankara 06230, Turkey; yas.oguz99@yahoo.com

**Keywords:** tularemia, *Francisella tularensis*, lipopolysaccharide, LPS, diagnostic, monoclonal antibodies, lateral flow immunoassay, enzyme-linked immunosorbent assay, patient samples, antibodies

## Abstract

*Francisella tularensis* is the causative agent of tularemia, a zoonotic bacterial infection that is often fatal if not diagnosed and treated promptly. Natural infection in humans is relatively rare, yet persistence in animal reservoirs, arthropod vectors, and water sources combined with a low level of clinical recognition make tularemia a serious potential threat to public health in endemic areas. *F. tularensis* has also garnered attention as a potential bioterror threat, as widespread dissemination could have devastating consequences on a population. A low infectious dose combined with a wide range of symptoms and a short incubation period makes timely diagnosis of tularemia difficult. Current diagnostic techniques include bacterial culture of patient samples, PCR and serological assays; however, these techniques are time consuming and require technical expertise that may not be available at the point of care. In the event of an outbreak or exposure a more efficient diagnostic platform is needed. The lipopolysaccharide (LPS) component of the bacterial outer leaflet has been identified previously by our group as a potential diagnostic target. For this study, a library of ten monoclonal antibodies specific to *F. tularensis* LPS were produced and confirmed to be reactive with LPS from type A and type B strains. Antibody pairs were tested in an antigen-capture enzyme-linked immunosorbent assay (ELISA) and lateral flow immunoassay format to select the most sensitive pairings. The antigen-capture ELISA was then used to detect and quantify LPS in serum samples from tularemia patients for the first time to determine the viability of this molecule as a diagnostic target. In parallel, prototype lateral flow immunoassays were developed, and reactivity was assessed, demonstrating the potential utility of this assay as a rapid point-of-care test for diagnosis of tularemia.

## 1. Introduction

Tularemia is a potentially lethal zoonotic disease caused by the intracellular Gram-negative bacterium *Francisella tularensis*. This organism is considered a Tier 1 select agent through the Federal Select Agent Program due to its low infectious dose, high mortality rates when not treated appropriately, and possibility of aerosolization [1,2]. *F. tularensis* has the potential to be easily disseminated and cause widespread illness and mortality, with estimates suggesting a large scale aerosol dispersal of 50 kg of bacteria over a population of 5 million could result in incapacitating casualties in 5% of the population [3,4]. Natural hosts include insects, mammals, birds and even fish, although the primary reservoir of infection is unknown [5]. Infection of humans can occur through many routes, such as arthropod vectors, direct contact with infected animals, water contamination and aerosol inhalation, thus presenting a rare but significant risk to public health [5,6,7,8]. Endemic areas primarily fall in the northern hemisphere, including North America, Europe and parts of Asia, with some studies indicating recent increases in the numbers of reported cases, particularly in northern Europe [9]. Both sporadic and clustered reported cases of tularemia have steadily increased in Germany since 2002 [10], and re-emerged in the Netherlands in 2013 after a 60 year period without reported isolation [11]. Geographical modeling has suggested that the increase in both tularemia cases and the range of geographical endemicity may be due to expansion of vector habitats as a result of climate change across Europe [12]. Currently, environmental surveillance relies on polymerase chain reaction (PCR) assays of soil and water samples [13], and isolation of bacteria from wildlife in affected areas, particularly small mammals [14,15].

Severity of human tularemia infection is dependent on several factors, including the strain and route of infection. There are three main subspecies: *tularensis, holarctica* and *mediasiatica* [16]. *F. tularensis* subspecies *tularensis* is the most virulent subspecies but is responsible for fewer naturally occurring infections worldwide. Type A strains such as *F. tularensis* subsp. *tularensis* are found primarily in North America [17]. Type B strains such as *F. tularensis* subsp. *holarctica* exhibit lower mortality but are responsible for the majority of naturally-acquired infections, predominantly in Europe and Asia [18]. There are no published accounts of the *mediasiatica* subspecies causing human disease [19].

Symptoms of infection are non-specific and vary greatly in severity. Tularemia presents most commonly as an acute febrile illness with symptoms such as fever, body aches and swollen lymph nodes [7]. However, presentation can include more varied symptoms depending on the route of infection, often complicating diagnosis. Respiratory tularemia resulting from inhalation of aerosolized *F. tularensis* is the most severe of the organ-specific infections, particularly involving Type A strains. Without rapid administration of the correct antibiotic therapy, the mortality rate for infection with this form can be as high as 60% [7]. It has been calculated that the infectious dose via the aerosol route could be as low as one bacterium based on animal models, underscoring the serious threat an aerosol release of this pathogen would present to public health [20]. Generally less severe forms of tularemia include ulceroglandular tularemia, wherein painless ulcers form at the site of infection [21,22], exposure of the eye (oculoglandular tularemia) or infection via contaminated food or water (oropharyngeal tularemia) [23].

Diagnosis of tularemia can be made definitively by direct culture from blood or ulcers, lymph node biopsies and sputum; however isolation from the blood in the early stages of infection is rare and challenging due to low circulating numbers of bacteria and the fastidiousness of the organism with regard to growth conditions [24]. Culture of *F. tularensis* can also take up to 10 days, an unacceptably long time for such a potentially serious illness, especially in the event of widespread exposure. Confirmation of tularemia diagnosis can be made by measuring the fold change in serological response to infection via detection of antibodies to *F. tularensis* lipopolysaccharide (LPS) in patient serum. This approach is limited in that often antibodies do not reach diagnostically significant levels until approximately two weeks post-infection [25], and can persist for decades particularly in endemic areas, thus potentially complicating later diagnosis and meaning that changes in titers over time need to be monitored [26]. Development of PCR-based assays for detection and diagnosis of tularemia have shown promise in terms of increased sensitivity when compared to culture techniques [27]. Unfortunately, these assays cannot be easily integrated at point-of-care and require complex lab equipment and skilled personnel to perform. There is currently no standardized point-of-care diagnostic for tularemia, and thus recognition of an outbreak or release would likely be entirely dependent on identification by a public health authority after more common infections had been ruled out [3]. The delay of diagnosis and therefore appropriate therapy could result in development of advanced pneumonia or sepsis and death. Thus, a simple, rapid and reliable diagnostic is needed, particularly for use in a mass exposure or outbreak setting [3].

LPS, also known as endotoxin, forms the majority of the Gram-negative bacterial cell envelope and is implicated in stimulation of the host immune response during bacterial infection [28]. LPS has three main structural components: lipid A, core region and O-antigen. *F. tularensis* LPS is atypical, primarily in that the lipid A component is tetraacylated with 16–18 carbon fatty acid chains, vs. the more prototypical lipid A which is hexaacylated with 12–14 carbon fatty acid chains [29]. Modification of this lipid A structure is thought to play a key role in the immune evasion strategy of *F. tularensis*, preventing stimulation of the TLR-4 pro-inflammatory pathway common during other bacterial infections [30]. The O-antigen component of LPS is widely regarded as the immunodominant region and is composed of a polysaccharide chain that can vary in length and the sugars present in the chain [31]. Structural characterization of the *F. tularensis* LPS O-antigen has shown that pathogenic Type A and Type B isolates have identical O-antigen structures, whereas *F. novicida*, a close relative of *F. tularensis* has antigenically distinct external carbohydrate residue structures [32], differences that are reflected in the O-antigen gene clusters of these organisms [33,34]. Studies have shown that O-antigen-like polysaccharides can also be found on the surface of *F. tularensis* in the form of a capsule, without the lipid A or core components of LPS [35].

Our laboratory previously identified LPS as a potential diagnostic antigen for tularemia in antigen discovery studies due to its reactivity with murine immune sera, abundance on the bacterial outer surface and potential to be shed at detectable levels [36,37]. Host antibodies against *F. tularensis* LPS have been detected in patient serum via Western blot [38] and enzyme-linked immunosorbent assay (ELISA) [39,40]. LPS itself has not been detected and quantified directly and no defined clinical range of LPS concentrations in patient samples has been suggested for diagnostic purposes. Direct detection of LPS from patient samples may serve as an earlier, more accurate diagnostic than currently available assays, especially if implemented at the point of care.

The goal of this study was to isolate a library of high-affinity monoclonal antibodies (mAbs) reactive with *F. tularensis* LPS for use in antibody-based diagnostics capable of detecting LPS in patient samples. Ten mAbs were isolated and reactivity with type A and type B strains of *F. tularensis* was determined. mAbs were evaluated in all pairwise combinations and top performing mAb pairs were integrated into (i) a highly sensitive antigen-capture ELISA for laboratory-based detection and quantification of *F. tularensis* LPS and (ii) a prototype lateral flow immunoassay (LFI) for rapid point-of-care diagnosis of tularemia. The antigen-capture ELISA was optimized for use with human matrices and used for quantification of LPS in tularemia patient samples. Prototype LFIs were constructed and reactivity with clinically relevant strains was determined.

## 2. Results

### 2.1. mAb Production and Reactivity

**Figure 1:**
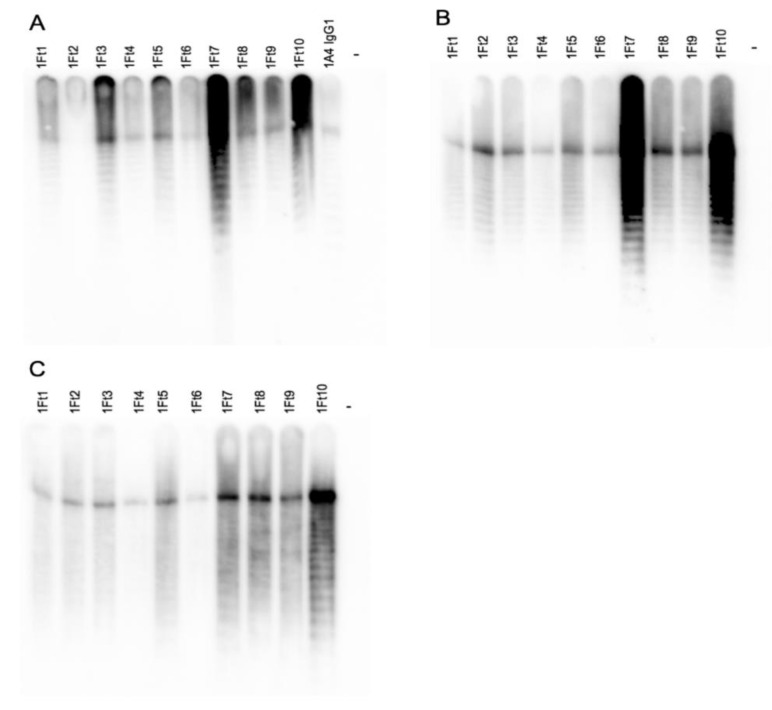
mAb reactivity with purified *F. tularensis LPS and F. tularensis* strains (type A and type B). Purified, HRP-conjugated mAbs were used to probe 1 μg/lane purified LPS from *F. tularensis* subsp. *holarctica* LVS. Previously isolated *F. tularensis* antibody 1A4 IgG1 was included as a positive control (**A**), 1 × 10^8^ CFU F. *tularensis* subsp. *tularensis* Schu S4 (type A strain) (**B**) and *F. tularensis* subsp. *holarctica* (FRAN-012) (type B strain) loaded across 11 wells (9.09 × 10^6^ cfu/lane) (**C**) by direct Western blot.

Ten mAb-producing hybridoma cell lines were created from female CD1 mice immunized with LPS purified from the CDC Live Vaccine Strain of *F. tularensis* subsp. *holarctica* (BEI Resources) conjugated to BSA and administered with or without Alhydrogel^®^ adjuvant 2% (alum). mAbs were purified and subclass was determined by indirect ELISA. A combination of IgG1 and IgG2b mAbs were isolated and characterized (Table 1). Western blots were performed using proteinase K treated killed cells to determine reactivity with *F. tularensis* subsp. *tularensis* strain Schu S4, *F. tularensis* subsp. *holarctica* (FRAN-012), *F. novicida* strain U112 and *Francisella philomiragia*, a near neighbor [41]. Representative blots showing reactivity of all ten mAbs plus previously isolated *F. tularensis* mAb 1A4 IgG1 [42] with purified LPS, and reactivity of the ten newly purified mAbs with pathogenic *F. tularensis* Schu S4 (Type A) and *holarctica* (FRAN-012) (Type B) are shown in Figure 1.

### 2.2. Antigen-Capture ELISA Optimization

**Figure 2:**
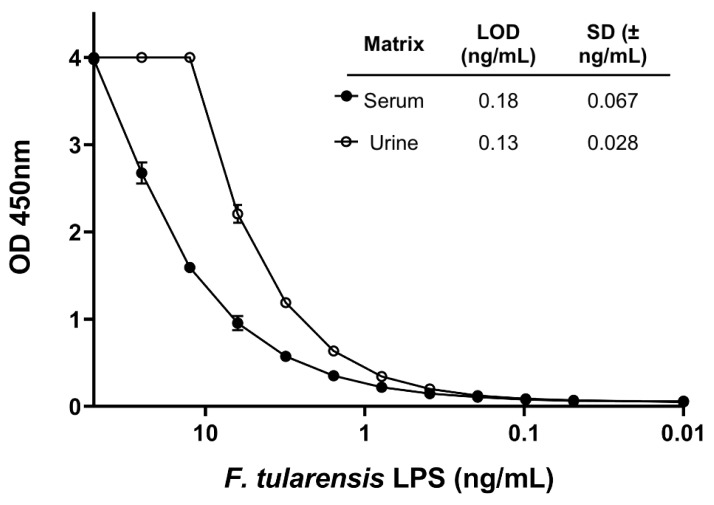
Sensitivity of optimized *F. tularensis* LPS antigen-capture ELISA. Limit of detection (LOD) of capture antibody 1Ft5 and detection antibody 1Ft7-HRP was assessed. The standard curve of the optimized antigen capture ELISA with *F. tularensis* LPS spiked into normal human serum and urine is shown. LOD of the assay in each matrix was calculated using a cutoff value of 2× background. LOD in serum was 0.18 ± 0.067 ng/mL and 0.13 ± 0.028 ng/mL (*n* = 3).

To develop a quantitative immunoassay for *F. tularensis* LPS, mAbs were HRP-conjugated and tested in antigen-capture ELISA format. Each mAb was tested in both the capture and detection position at a standard concentration of 1 μg/mL diluted in PBS for the capture or blocking buffer for the detection. The cut-off OD value used to determine a positive test for a given antibody pair, or limit of detection (LOD), for LPS in PBS was calculated at 3× background OD 450 nm value (no antigen) and an average of two rows taken to rank the pairs to proceed with optimization (Table S1). The importance of developing a panel of mAbs to test different pairs can be seen in the variation between pairs shown in Table S1. Two pairs (1Ft5-capture, 1Ft7-HRP and 1Ft9-capture and 1Ft7-HRP) were selected for the lowest consistent LOD, and antibody coating and HRP conjugate concentrations were then optimized in pooled normal human serum and urine spiked with *F. tularensis* LPS to determine the LOD in relevant matrices and the most sensitive pairing selected. Optimization in clinically relevant samples is important as these matrices can affect assay performance differently compared to buffer alone. Antibodies 1Ft5 (capture): 1Ft7-HRP (detection) were selected as the optimal pairing at a concentration of 2.5 μg/mL coating and 0.625 μg/mL HRP. These conditions gave an LOD of 0.18 ng/mL in normal human serum and 0.13 ng/mL in normal human urine, the standard curves for which are shown in Figure 2. LOD was calculated at 2× background in triplicate.

### 2.3. Quantification of LPS in Patient Samples

Archived tularemia patient serum samples collected between February 2010 and January 2012 were obtained from Hacettepe University and 0.2 μm filtered at the University of Nevada Reno to remove any viable bacteria in a BSL-3 laboratory. Samples were verified for sterility using a validated procedure, which allowed for analysis under BSL-2 conditions. Nineteen samples were of sufficient volume for analysis by antigen-capture ELISA for the presence of shed LPS. LPS was detected in eight of the samples and the concentration calculated by comparison to a standard curve of purified LPS. As shown in Table 2, the concentration ranged from 0.22 ng/mL to 109.95 ng/mL. The proximity of the calculated concentrations to the LOD of the ELISA in many of these samples may indicate that additional negative samples may contain LPS below quantifiable levels with this assay. Samples were analyzed in triplicate unless otherwise indicated due to sample volume limitations. The number of days between noticing lymph node enlargement and sample collection is shown to provide an indication of the stage of infection, however as these samples are from naturally occurring infections it is difficult to accurately determine the time from infection. Positive samples range from 4–30 days post notice of lymph node enlargement, and negative samples from 2–60 days. It is possible that there is a window for LPS detection between this range, however more samples are needed to determine whether this is the case. It was also reported that 52.9% of these patients received antimicrobial treatment prior to sample collection, which may have affected circulating bacteria levels.

### 2.4. LFI Development

To evaluate the potential of the isolated antibodies in an LFI format for development into a rapid diagnostic test, all ten mAbs were evaluated in both the capture and detection position for reactivity with purified *F. tularensis* LPS, along with 1A4 IgG1, an anti-*F. tularensis* LPS antibody isolated in a previous study [42]. Initial evaluation of LFIs involved testing with a standard concentration of LPS in buffer compared to a control of buffer alone. Visual assessment of test line signal intensity was performed for each mAb pairing. In addition, analysis with a Qiagen ESE-Quant lateral flow reader was performed in order to quantify test line intensity and non-specific binding at the test line when LPS was not present in the sample to facilitate ranking of pairs. Details of this testing are in Table S2. Criteria such as signal minus background with and without a blocking agent at a standard concentration of 500 ng/mL LPS followed by a preliminary limit of detection for each prototype to indicate potential sensitivity at lower concentrations resulted in a ranking system to isolate the top 20 pairs out of a possible 121 combinations. The top 20 were further tested with varying concentrations of casein as a blocking agent to optimize blocking and reduce non-specific binding. Pairs were tested for a preliminary limit of detection with the optimized casein concentration and ranked to proceed into further testing and optimization.

A prototype LFI was developed using mAb 1Ft6 immobilized on the test line and 1Ft5 as the gold conjugate following further testing and optimization of the top 20 LFIs, including testing for non-specific binding (false positives) in pooled normal human serum. This prototype was selected for its sensitivity and low levels of non-specific binding in buffer and normal human serum. LFI buffer conditions and components were optimized to increase sensitivity and reduce non-specific binding at the test line.

**Figure 3:**
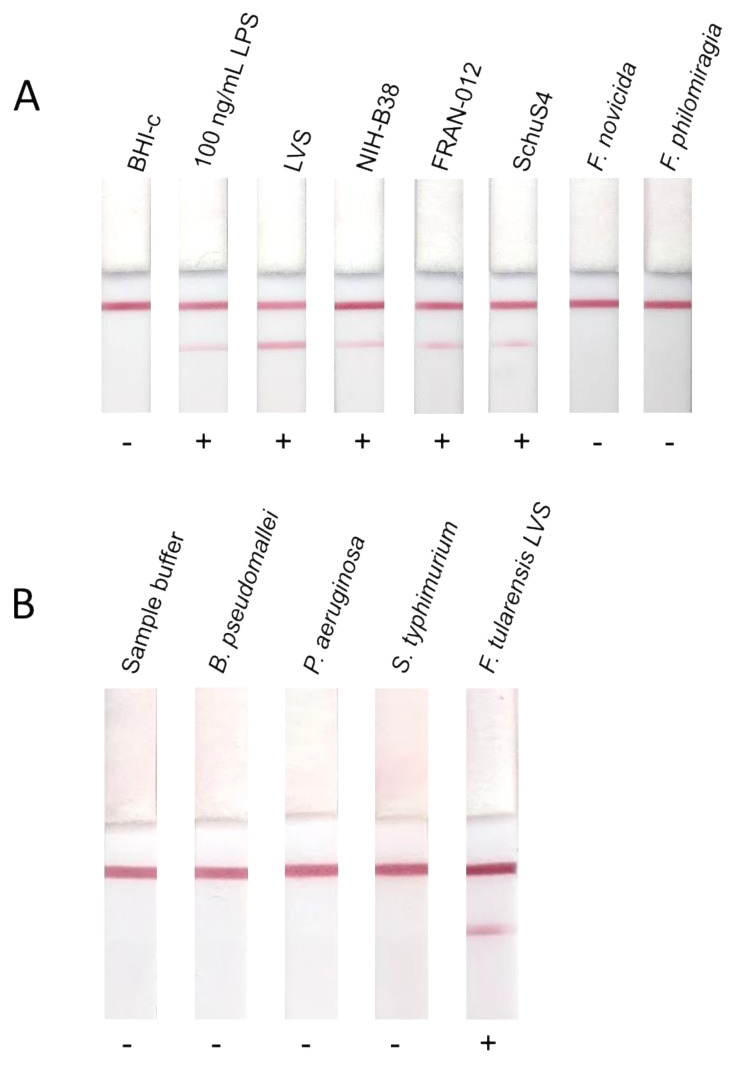
Reactivity of LFIs with clinically relevant *F. tularensis* strains and near neighbors, and purified LPS from different bacteria. Prototype LFIs were run with a panel of killed whole cells to determine potential usefulness as a diagnostic for tularemia. Brain Heart Infusion broth supplemented with cysteine (BHI-c) is included as a negative control. 100 ng/mL *F. tularensis* subsp. *holarctica* LVS LPS was included as a positive control. Whole cells from *F. tularensis* subsp. *holarctica* LVS (LVS), *F. tularensis* subsp. *tularensis* NIH-B38 (NIH-B38), *F. tularensis* subsp. *holarctica* isolate FRAN-012 (FRAN-012), *F. tularensis* subsp. *tularensis* Schu S4 (SchuS4), *F. novicida*, *F. philomiragia* were also tested for reactivity (**A**). Prototype LFIs were also tested with 100 ng/mL purified LPS from other species of bacteria to determine potential for cross-reactivity (**B**).

This LFI prototype was used to assess reactivity with purified *F. tularensis* LPS, heat inactivated *F. tularensis* live vaccine strain (LVS), heat inactivated *F. tularensis* strain NIH-B38, formalin inactivated *F. tularensis* subsp. *tularensis* Schu S4, gamma-irradiated *F. tularensis* subsp. *holarctica* (FRAN-012)*,* gamma-irradiated *F. novicida* U112 and gamma-irradiated *F. philomiragia.* The purpose of this testing was to determine reactivity of the prototype assay with 1 × 10^7^ CFU/mL for both type A and B strains, including BSL-2 (LVS and NIH-B38) and BSL-3 (Schu S4 and *holarctica)* strains as well as *F. novicida,* which has been shown to have a different LPS structure [34] and near neighbor *F. philomiragia.* Reactivity was observed with purified LPS and cells from both variants of the type A and B strains tested. No reactivity was observed with *F. novicida* or *F. philomiragia,* as expected due to differences in LPS structure and associated virulence. Representative LFIs are shown in Figure 3. Patient samples were not run on the LFI prototype due to limited sample volume, however whilst the full range of circulating LPS concentrations is unknown, the ELISA data provides a promising target for rapid detection. To begin to explore the specificity of the LFI, the prototype was tested using purified LPS from some other gram-negative bacteria and no false positive results were observed (Figure 3).

**Figure 4:**
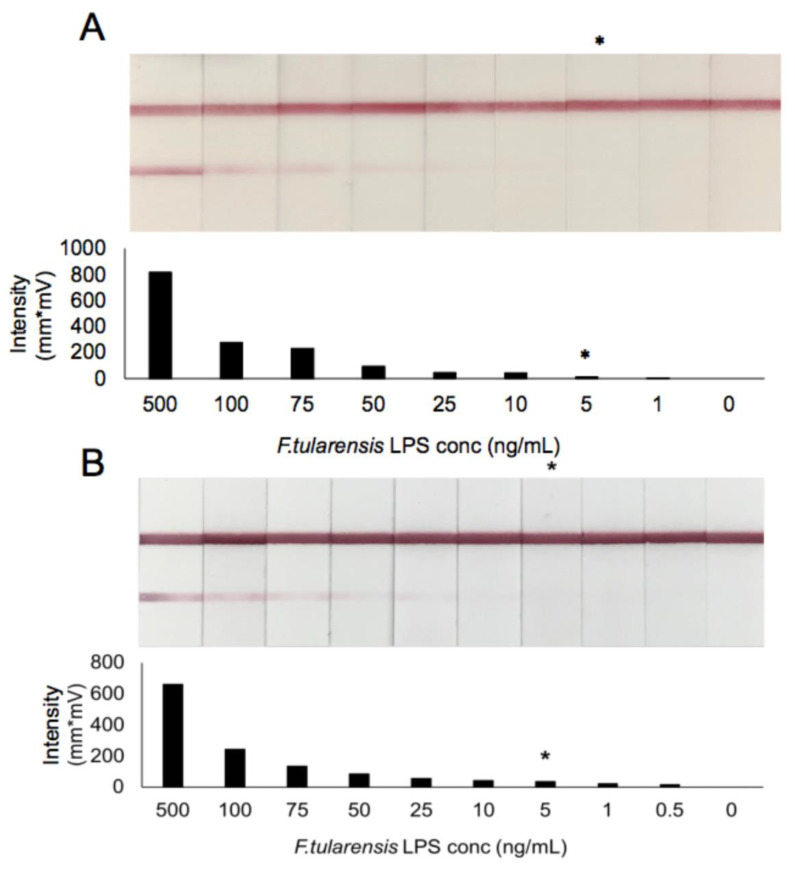
LFI prototype sensitivity in pooled normal human serum (**A**) and urine (**B**) spiked with purified *F. tularensis* LPS. Visual LOD is indicated (*) as assessed by three blinded readers. Test line intensity is shown as given by the ESE-Quant lateral flow reader.

Purified LPS was serially diluted in pooled normal human serum and urine to provide an indication of the potential sensitivity of the assay in patient matrices. LFIs were assessed visually by three blinded readers and the limit of detection taken as the lowest concentration detectable by all three. In addition to visual assessment, the LFIs pictured in Figure 4 were read using the Qiagen ESE lateral flow reader to provide a quantitative representation of binding. Visual examples of the dilution series in both serum and urine as well as the corresponding intensity of the signal for each test strip are shown in Figure 4. Based on this testing, the sensitivity of this assay in pooled normal human serum and urine was determined to be ~5 ng/mL. Further optimization of the assay for detection in these matrices can be done to potentially increase the sensitivity to the levels indicated in the ELISA analysis of the patient samples outlined above now that it is clear that the assay platform is functional in human matrices.

## 3. Discussion

When attempting to diagnose an infection with the range of clinical symptoms and potential fatality rates associated with tularemia, it is essential to make a quick and definitive diagnosis. This need is compounded by the status of *F. tularensis* as a potential biothreat. In the event of widespread exposure, or exposure in a combat situation, a rapid and accurate diagnostic will result in lives saved. Accessible assays for direct detection of *F. tularensis* LPS may also be useful for both field and laboratory analysis of environmental and wildlife samples for monitoring reservoirs of disease. Production of a library of novel antibodies specific to *F. tularensis* LPS allows for development of a sensitive and specific lateral flow immunoassay that could potentially be developed into a rapid point of care diagnostic for tularemia, a necessity for diagnosis and efficient resource allocation for treatment. We have shown that the prototype LFI is reactive with both *F. tularensis* subsp. *holarctica* and *F. tularensis* subsp. *tularensis* in both attenuated and fully virulent strains, whilst not reacting with near neighbors. This is an important step towards ensuring that a future diagnostic assay will be specific for tularemia.

Production of a monoclonal antibody library enables the development of new assays for diagnosis and potentially treatment of tularemia. All mAbs produced in this study were shown to be reactive by Western blot with both *F. tularensis* subsp. *tularensis* and *F. tularensis* subsp. *holarctica*, the strains responsible for causing the majority of human disease. In order to produce the most analytically sensitive (lowest LOD) ELISA and LFI it was crucial to test all mAbs in the capture and detection position to determine the best pair of mAbs for each specific assay. This all-by-all testing procedure is labor intensive, however facilitates the development of the most analytically sensitive assay possible, which can be seen in the variation in sensitivity in the all-by-all testing. Interestingly, although many mAb pairs were able to detect LPS with relative sensitivity, three mAbs were consistently amongst the most sensitive: 1Ft5 and 1Ft6 in the LFI, 1Ft5 and 1Ft7 in the ELISA. mAb pairs were finalized in their respective assays and conditions optimized in patient matrices of interest.

Our previous study that utilized a technique called In vivo Microbial Antigen Discovery (InMAD) to discover novel bacterial antigens supports the finding that LPS is shed/secreted into the blood during infection, as LPS reactivity was seen in immune serum from mice immunized with 0.2 µm filtered serum from a tularemia infection model [36]. Very little is known regarding the presence and concentration of soluble *F. tularensis* LPS within clinical samples, primarily due to a focus on detection of patient anti-LPS antibodies for diagnosis. It was therefore critical to utilize the antigen-capture ELISA to quantify LPS levels in tularemia patient serum samples as a starting point. LPS was quantifiable in 8/19 samples, a promising result given that these samples were filtered to remove viable bacteria, therefore any cell associated LPS was lost, and approximately half of the patients had received antibiotic treatment. It is also possible that freezing and thawing the samples prior to filtration may have resulted in lysis of bacterial cells and therefore more LPS available for detection in the filtered samples.

The abundance of LPS on the bacterial surface suggests that it may be a valuable diagnostic antigen. The quantifiable presence in filtered patient samples indicates a portion of LPS was shed/secreted into the blood during infection. Although LPS was detected in patient samples for this study, many of the concentrations are near the calculated limit of detection for the assay. It is possible that this low concentration is due to the loss of cell associated LPS, however it could also be true that serum is not the optimal matrix for detection of this antigen. Previous work from our group studying the shedding of *Burkholderia pseudomallei* capsular polysaccharide (CPS) has determined that the highest concentrations of CPS can be found in the urine, possibly making it the optimal diagnostic matrix for CPS detection [43]. It has been shown that *F. tularensis* can colonize the kidney in animal models and infected wild animals, therefore it is possible that LPS may also be shed into the urine [44,45,46,47]. Further studies are needed in order to determine if LPS is detectable in additional matrices such as urine, lymph node biopsies and abscesses, the latter of which are commonly reported to have high bacterial burden. Analysis of lymph node biopsies or aspirates is a common technique for confirming *F. tularensis* infection [48,49]. In addition to exploring presence of LPS in clinical samples, we would like to examine how LPS is shed/secreted over the course of *F. tularensis* infection in order to characterize how this biomarker can be most efficiently detected. The ELISA developed here is a useful tool that can be employed in future studies for establishment of a clinically relevant range in patient matrices and also in samples collected from animal models of tularemia.

We have shown that the prototype LFI is reactive with both *F. tularensis* subsp. *holarctica* and *F. tularensis* subsp. *tularensis* in both attenuated and fully virulent strains, whilst not reacting with near neighbors. The resulting LFI prototype is also non-reactive with purified LPS from several other bacterial species. The next step in assay development for commercialization is a more exhaustive cross-reactivity panel with other microbes that have similar clinical presentations or are commonplace amongst the population. Testing cross-reactivity is important to ensure that the test is specific for tularemia and reduce the likelihood of a false positive test.

When testing the sensitivity of the selected LFI prototype in human serum and urine as two potential matrices of interest, we were able to detect concentrations of LPS down to ~5 ng/mL as determined by three blinded readers. This is not yet sensitive enough to detect the LPS concentrations quantified in 6/8 samples by ELISA, however it does indicate that this antibody pair is functional in human matrices. It is again important to note that these samples were filtered and therefore do not contain any LPS associated with the bacterial cell and are likely not representative of LPS levels in an unfiltered sample. Furthermore, reduction in sensitivity of an assay prototype when moving from buffer to patient samples is not uncommon, as these samples differ in areas such as protein concentration and competing host antibodies [50]. As the assay tested is an early prototype, there are many areas of the assay that can be optimized to better accommodate different sample types. Examples include addition of sample pads treated to buffer samples before they reach the nitrocellulose, or additives to the sample or running buffers to neutralize the effects of excess proteins in the sample to be tested [51]. Lateral flow assays have the potential to be able to accommodate many different sample types through optimization of components and minor changes to sample preparation protocols [52,53]. Further optimization of this prototype alongside the potential incorporation of these antibodies in other platforms for detection of *F. tularensis* LPS could lead to development of more effective diagnostics for tularemia.

## 4. Materials and Methods

### 4.1. mAb Production

8-week-old female CD1 mice (Charles River Laboratories, Inc., Wilmington, MA, USA) were immunized intraperitoneally with purified *F. tularensis* subspecies *holarctica* LVS LPS (NR-2627) (BEI Resources, Manassas, VA, USA) coupled to BSA using the Imject™ EDC BSA Spin Kit (Thermo Scientific, Waltham, MA, USA) to improve immunogenicity. Immunizations were performed with and without Alhydrogel^®^ adjuvant 2% (Invivogen, San Diego, CA, USA). Mice in both conditions were immunized with 10 μg of *F. tularensis* LPS-BSA subcutaneously and a further 10 μg given at 6 and 8 weeks post initial immunization. Boosts of 25 μg of *F. tularensis* LPS-BSA were given at weeks 11 and 13 post immunization. An indirect ELISA was used as outlined below to determine antibody titers to LPS in mouse immune serum. Mice were immunized with a final dose of 5 μg of purified LPS-BSA three days prior to spleen harvest. Fusions were performed using P3x63Ag.653 fusion partner and hybridoma cells produced using standard techniques [54]. Supernatant was collected from hybridoma cells and mAbs purified using recombinant protein A affinity chromatography.

### 4.2. Ethics Statement

Laboratory work with animals was approved by the University of Nevada, Reno Institutional Animal Care and Use Committee (Protocol # 00024). All work with animals is supervised by the Office of Laboratory Animal Medicine, which follows the National Institutes of Health Office of Laboratory Animal Welfare policies (Assurance # A3500-01).

### 4.3. Indirect ELISA

96-well medium-binding microtiter plates (Grenier Bio-One, Kremsmünster, Austria) were coated with 1.25 μg/mL of *F. tularensis* LPS overnight. The plates were then washed 3x with PBS containing 0.05% Tween 20 (PBS-T) and blocked for 90 min at 37 °C in PBS containing 0.5% non-fat milk and 0.1% Tween 20 (blocking buffer), followed by a second wash in PBS-T. Primary antibody in the form of mouse immune serum, hybridoma supernatant or purified antibody (1 μg/mL) was added to the first well and serial two-fold dilutions performed across the plate. The plate was incubated at room temperature for 1 h. The plate was then washed with PBS-T and incubated with horseradish peroxidase (HRP) labeled goat anti-mouse IgG antibody (SouthernBiotech, Birmingham, AL, USA), either whole IgG or isotype specific, at a 1:1000 dilution in blocking buffer for 1 h at room temperature. The plate was washed a final time in PBS-T and incubated with tetramethylbenzidine (TMB) substrate (SeraCare, Milford, MA, USA) for 30 min. The reaction was stopped with 1M H_3_PO_4_ and the absorbance read at OD_450_.

### 4.4. Western Immunoblot

Standard semidry Western blot procedure was performed using 1 μg/lane purified *F. tularensis* subspecies *holarctica* LVS LPS (NR-2627) (BEI Resources) or proteinase K-treated 5 × 10^8^ CFU/mL of formalin inactivated *F. tularensis* subsp. *tularensis* strain Schu S4 cells (NR-15753) (BEI Resources) or using 5 × 10^8^ CFU/mL of gamma-irradiated *F. tularensis* subsp. *holarctica* cells (FRAN-012), *F. novicida* (FRAN-003) and *F. philomiragia* (FRAN-017) (Department of Defense Critical Reagents Program, Frederick, MD, USA). 1 × 10^8^ cfu of each bacteria were loaded in 200 μL across 11 wells, providing 9.09 × 10^6^ cfu/lane. Samples were separated on 10% SDS gel (Bio-Rad Laboratories, Hercules, CA, USA) and transferred to nitrocellulose membrane (Bio-Rad). HRP-conjugated mAbs 1Ft1-10 were used to probe the membrane at a concentration of 1 μg/mL using a Miniblotter system (Interchim, Montluçon, France), which enables probing of one antigen preparation with multiple antibodies. Signal was detected with SuperSignal™ West Femto Maximum Sensitivity Substrate (Thermo Fisher Scientific, Waltham, MA, USA). Images were taken using a ChemiDoc XRS system (Bio-Rad).

### 4.5. Antigen-Capture ELISA

96-well microtiter plates were coated with 100 µL/well of capture mAb (1 µg/mL) in PBS overnight. Plates were washed with PBS-T and blocked at 37 °C with 200 µL/well of blocking buffer. Purified LPS was added to the first well at a concentration of 100 ng/mL and serial diluted two-fold across each plate in blocking buffer for a final volume of 100 µL/well. Plates were incubated for 60 min at room temperature, then washed with PBS-T and incubated with HRP-labelled mAb at 1 µg/mL in blocking buffer for a total of 100 µL/well. HRP labelling of mAbs was done using EZ-link Plus Activated Peroxidase (ThermoFisher, Waltham, MA, USA). Plates were washed with PBS-T and incubated with 100 µL/well of TMB substrate (SeraCare). The reaction was stopped after 30 min with 1M H_3_PO_4_ (100 µL/well). Plates were read at an optical density of 450 nm (OD_450_).

### 4.6. Optimization of Antigen-Capture ELISA in Serum and Urine

Checkerboard ELISAs were performed to optimize the concentrations of coating and detection mAbs in pooled normal human serum and urine (Innovative Research, Novi, MI, USA). Capture and detection mAb concentrations were both tested at a range of concentrations from 0.16–20 μg/mL to assess which concentration was the most sensitive without exhibiting non-specific binding. The remainder of the ELISA was performed as described above, with purified *F. tularensis* LPS spiked into pooled normal human serum or urine at a concentration of 50 ng/mL, serially diluted in blocking buffer and incubated for 90 min at room temperature. Final optimized conditions were chosen for 1Ft5 capture (2.5 μg/mL) and1Ft7 detection (0.625 μg/mL) as the pair that gave the lowest LOD in both serum and urine for potential future diagnostic applications.

### 4.7. Patient Samples

Archived samples from patients with confirmed diagnosis of tularemia either by serological or PCR techniques were obtained from Hacettepe University (Ankara, Turkey). The samples were collected between February 2010 and January 2012 and stored at −80 °C until they were sent to the University of Nevada, Reno in 2018. Experiments using human samples were approved by the University of Nevada, Reno Institutional Review Board. Samples were received at biosafety level 3 and 0.2 μm filtered to remove viable bacteria. Each sample was verified for sterility using a validated protocol wherein 10% of each sample was placed in brain heart infusion broth supplemented with cysteine and incubated for 72 h at 37 °C. 100 μL of each broth was then plated onto cystine heart agar and incubated at 37 °C for 9 days. Plates were examined for growth and removed to biosafety level 2 for analysis if no growth was observed.

### 4.8. Quantitative Antigen-Capture ELISA

An optimized antigen capture ELISA was performed using the tularemia patient serum samples according to the optimized conditions described above with mAb 1Ft5 coated in PBS at 2.5 μg/mL overnight. Plates were washed and blocked, then purified *F. tularensis* LPS (BEI Resources) was two-fold serially diluted across the plate starting at 50 ng/mL as a standard curve, totaling 100 μL/well. 200 μL of patient serum samples were added to the plate and 2-fold serial diluted across prior to incubation for 2 h at room temperature. Plates were washed again and 100 μL/well of HRP-conjugated 1Ft7 was added at 0.625 μg/mL diluted in blocking solution for 1 h. Plates were washed and incubated with 100 μL/well of TMB substrate for 30 min (SeraCare). The reaction was stopped with 100 μL/well of 1M H_3_PO_4_ and read at OD_450_. Samples were analyzed in triplicate where possible, however due to limited sample volumes available this was not feasible for all samples.

### 4.9. LFI Screening

Initial screening was performed with each mAb in the capture position on the test line and as the detection gold conjugate to test every combination and rank the most sensitive pairings. Testing was done using a default LFI prototype to test reactivity to purified LPS in PBS and non-specific binding in buffer alone. Briefly, 5 μL of gold conjugate at OD 10 was added to the conjugate pad, followed by 40 μL of 500 ng/mL LPS in PBS. The test was then placed vertically in the well of a microtiter plate containing 150 μL of chase buffer and allowed to run for 15–20 min. LFIs were evaluated visually and read using an ESE-Quant lateral flow reader (Qiagen, Hilden, Germany), then ranked based on the intensity of the test line minus non-specific binding in buffer alone. The best performing pairs were also tested in 1% casein, the percentage of which was then optimized to determine the potential for addition of blocking reagents to reduce non-specific binding and impact signal. Pairs were then ranked according to the LOD with optimized casein conditions. The top 20 candidates were tested similarly in pooled normal human serum to select the pair with best signal and lowest non-specific binding in human matrices for downstream application.

### 4.10. LFI Prototype

Upon selection of 1Ft6 as the optimal capture mAb and 1Ft5 as the gold conjugated mAb according to the above selection criteria, optimization of LFI components and reagents was undertaken. Primary areas of optimization included testing of different surfactants and blocking agents in the gold conjugate diluent and sample buffer, as well as testing nitrocellulose membranes with different flow rates and treatments for improved sensitivity. The optimized conditions selected to proceed were as follows: 1Ft6 was applied to CN95 nitrocellulose membrane (Sartorius, Gottingen, Germany) at a concentration of 1 mg/mL in PBS as the test line via contact dispense using a BioDot XYZ platform (BioDot, Irvine, CA, USA). Goat anti-mouse Ig (SouthernBiotech) was dispensed as the control line at 0.5 mg/mL also in PBS. Nitrocellulose was dried for 30 min at 37 °C. LFIs were assembled onto an adhesive backing card with the sprayed nitrocellulose overlapped by CF6 wicking pad (GE Healthcare, Chicago, IL, USA) to allow for capillary flow. Test strips were cut to 5 mm width and stored in sealed foil pouches with desiccants. 1Ft5 was passively adsorbed to 40 nm colloidal gold particles (DCN Diagnostics, Carlsbad, CA, USA), and diluted to OD_540_ = 10 in an optimized buffer containing 0.05M sodium phosphate, 0.2% surfactant 10G (Fitzgerald Industries International, Acton, MA, USA), 0.25% BSA, 20% sucrose and 5% trehalose.

### 4.11. LFI Testing

LFI prototypes were tested with inactivated cells from various strains of *F. tularensis* and near neighbors to confirm reactivity with clinically relevant *F. tularensis* strains and their derivatives and to ensure no cross-reactivity with near neighbors known to have structurally distinct LPS. Glycerol stocks of *F. tularensis* subspecies *holarctica* LVS and *F. tularensis* subspecies *tularensis* NIH-B38 (BEI Resources) were grown in BHI broth supplemented with cysteine and inactivated by heating to 80 °C for two hours. OD_600_ was taken and the preparations diluted to approximately 1 × 10^7^ CFU/mL. Formalin inactivated *F. tularensis* subsp. *tularensis* strain Schu S4 (BEI Resources), gamma-irradiated *F. tularensis* subsp. *holarctica* (FRAN-012) cells, *F. novicida* and *F. philomiragia* (Critical Reagents Program) were diluted to 1 × 10^7^ CFU/mL based on the product information provided. BHI broth supplemented with cysteine spiked into sample buffer (50 mM borate, 0.5% BSA, 1 μg/mL Mouse IgG, 1% surfactant 10G) and sample buffer alone were used as the negative controls. Samples were tested by placing the strip in a 96 well plate containing 18 μL of sample buffer and 2 μL of antigen. Once all liquid in the well was absorbed, the strip was moved to a well containing 15 μL of sample buffer and left until all buffer was absorbed. The strip was then moved to a well containing 15 μL of sample buffer and 5 μL of 1Ft5 gold conjugate at OD_540_ = 10. Once all gold was absorbed, the strip was moved to a final well containing 40 μL of sample buffer. Once all liquid was absorbed the LFI was assessed visually for reactivity as it would be by a clinician. To test reactivity to purified LPS, the above procedure was followed but with 100 ng/mL of purified LPS from *B. pseudomallei, Pseudomonas aeruginosa*, *Salmonella typhimurium* (Obtained from Dr Paul Brett at the University of Nevada, Reno) and 100 ng/mL *F. tularensis* LPS (BEI Resources) instead of inactivated cells. To determine assay sensitivity in pooled normal human serum and urine, the same procedure outlined above was followed but with 18 μL total volume serum or urine containing 100 ng/mL mouse IgG and 1% surfactant 10G and 2 μL of purified *F. tularensis* LPS. Signal was assessed as positive or negative by three blinded readers and the LFIs read on an ESE-Quant lateral flow reader (Qiagen, Hilden, Germany).

## Figures and Tables

**Table 1:** IgG subclass and immunization strategy of mAbs. Immunizations were *F. tularensis* LPS conjugated to BSA with or without Alhydrogel^®^ adjuvant 2% (alum).

mAb	Subclass	Immunization
1Ft1	IgG1	Ft. LPS-BSA
1Ft2	IgG2b	Ft. LPS-BSA
1Ft3	IgG2b	Ft. LPS-BSA
1Ft4	IgG1	Ft. LPS-BSA + Alum
1Ft5	IgG1	Ft. LPS-BSA
1Ft6	IgG2b	Ft. LPS-BSA + Alum
1Ft7	IgG2b	Ft. LPS-BSA + Alum
1Ft8	IgG2b	Ft. LPS-BSA + Alum
1Ft9	IgG2b	Ft. LPS-BSA + Alum
1Ft10	IgG2b	Ft. LPS-BSA + Alum

**Table 2:** Analysis of LPS concentrations in patient serum samples by antigen-capture ELISA (*n* = 3 unless otherwise indicated). Time since lymph node enlargement was noticed at collection of sample is provided as an indicator of the stage of infection where available (ND, not determined).

Sample #	LPS (ng/mL)	Standard Deviation	Diagnosis	Time Since Lymph Node Enlargement (Days)
1	0	-	PCR	30
2	Insufficient volume	-	PCR	ND
3	0	-	PCR	4
4	Insufficient volume	-	PCR	20
5	0	-	PCR	2
6 ^#^	0.74	0.0039	PCR	4
7	0	-	PCR	2
8	0	-	PCR	2
9 *	0.35	-	PCR	6
10	0.22	0.044	PCR	3
11	109.95	12.11	PCR	5
12	0.41	0.088	PCR	ND
13	0	-	PCR	ND
14	0	-	Serology	60
15	0	-	Serology	20
16	0	-	Serology	ND
17	0	-	Serology	17
18	5.023	0.70	Serology	30
19	0.36	0.051	Serology	21
20	0.33	0.16	Serology	15
21	0	-	Serology	20

* Sample analyzed as a single replicate due to sample volume limitations. ^#^ Sample analyzed in duplicate due to sample volume limitations.

## Data Availability

Data is contained within the article and supplementary material.

## Supplementary Materials

The following are available online at https://www.mdpi.com/article/10.3390/pathogens10080924/s1: Table S1: Preliminary sensitivity of mAb pairs evaluated in an antigen-capture ELISA with *F. tularensis* LPS antigen (ng/mL) spiked into PBS determined by limit of detection pre-optimization. Pairs chosen to proceed with optimization are highlighted in bold. Table S2: Ranking of potential LFI pairs. Initial testing and ranking of top 20 mAb pairs in the LFI format to determine the optimal combination to proceed with further optimization. Casein percentage in the running buffer was optimized to rank pairs based on LOD with reduced non-specific binding prior to optimization of blocking conditions.
