# Supplementary material for: Development of Immunoassays for Detection of Francisella tularensis Lipopolysaccharide in Tularemia Patient Samples
Source: Pathogens. 2021 Jul 22;10(8):924. doi: 10.3390/pathogens10080924 (PMC8401977; doi:10.3390/pathogens10080924)
Supplement: Supplementary file 1 [file pathogens-10-00924-s001.zip › Table S1.pdf]

Table S1. Preliminary sensitivity of mAb pairs evaluated in an antigen-capture ELISA with *F. tularensis* LPS antigen (ng/mL) spiked into PBS determined by limit of detection pre-optimization. Pairs chosen to proceed with optimization are highlighted in bold.

		Detection mAb[a]									
		1Ft1	1Ft2	1Ft3	1Ft4	1Ft5	1Ft6	1Ft7	1Ft8	1Ft9	1Ft10
Capture mAb	1Ft1	2.43	0.56	1.17	17.40	4.81	3.43	1.00	1.54	0.81	12.98
	1Ft2	1.11	1.47	8.35	13.63	1.98	0.54	0.42	1.28	0.80	17.44
	1Ft3	0.61	2.38	0.50	14.83	3.78	1.74	1.18	1.82	1.77	16.72
	1Ft4	7.22	11.53	6.48	52.39	15.36	8.24	5.56	8.61	6.31	91.93
	1Ft5	0.93	2.61	0.89	15.47	3.74	1.49	**0.39**	0.46	0.68	3.92
	1Ft6	1.69	0.92	16.25	8.92	3.1	0.97	0.98	1.18	0.68	9.39
	1Ft7	0.53	0.99	0.71	13.59	0.47	0.49	0.41	0.45	0.48	2.31
	1Ft8	0.97	0.96	0.79	10.03	2.72	0.95	0.35	0.63	0.40	3.88
	1Ft9	1.97	1.14	0.92	19.96	4.45	0.57	**0.45**	0.54	0.37	5.11
	1Ft10	4.34	4.91	3.98	56.50	20.37	1.71	0.96	4.15	0.94	13.12

[a] Each mAb was used at a standard concentration of 1 μg/mL for both capture and detection
