# Supplementary material for: Development of Immunoassays for Detection of Francisella tularensis Lipopolysaccharide in Tularemia Patient Samples
Source: Pathogens. 2021 Jul 22;10(8):924. doi: 10.3390/pathogens10080924 (PMC8401977; doi:10.3390/pathogens10080924)
Supplement: Supplementary file 1 [file pathogens-10-00924-s001.zip › Table S2.pdf]

Table S2. Ranking of potential LFI pairs. Initial testing and ranking of top 20 mAb pairs in the LFI format to determine the optimal combination to proceed with further optimization. Casein percentage in the running buffer was optimized to rank pairs based on LOD with reduced non-specific binding prior to optimization of blocking conditions.

Capture	Gold	PBS signal - background	1% casein in PBS signal – background	Optimized casein %	LOD (ng/mL)
1Ft6	1Ft5	597	714	1.0	1
1Ft6	1Ft1	654	679	0.8	1
1Ft2	1Ft5	107	592	0.4	1
1Ft3	1Ft6	361	581	0.6	1
1Ft5	1Ft2	391	545	0.8	1
1Ft3	1Ft2	381	406	0.2	1
1Ft7	1Ft1	699	768	0.6	5
1Ft2	1Ft1	332	625	0.4	5
1A4	1Ft2	296	569	0.4	5
1Ft3	1Ft3	395	684	0.2	10
1Ft5	1Ft5	394	573	0.4	10
1Ft4	1Ft5	293	479	0.8	10
1Ft5	1Ft3	249	448	0.4	10
1Ft3	1Ft5	342	365	0.2	10
1A4	1Ft5	181	108	1.0	10
1Ft3	1Ft1	526	719	0.2	25
1Ft5	1Ft1	494	484	0.2	25
1A4	1Ft3	419	311	0.4	25
1Ft1	1Ft1	131	415	0.2	50
1A4	1Ft6	330	132	0.6	50
